# Association of serum surfactant protein D and SFTPD gene variants with asthma in Danish children, adolescents, and young adults

**DOI:** 10.1002/iid3.560

**Published:** 2021-11-15

**Authors:** Benjamin Hoffmann‐Petersen, Raymond Suffolk, Jens J. H. Petersen, Thomas H. Petersen, Charlotte Brasch‐Andersen, Arne Høst, Susanne Halken, Grith L. Sorensen, Lone Agertoft

**Affiliations:** ^1^ Hans Christian Andersen Children's Hospital Odense University Hospital Odense Denmark; ^2^ Open Patient Data Explorative Network Odense University Hospital Odense Denmark; ^3^ Institute of Clinical Research, Faculty of Health Sciences University of Southern Denmark Odense Denmark; ^4^ Department of Pediatrics Hospital of Southern Jutland Aabenraa Denmark; ^5^ Department of Pediatrics Hospital of Southern Jutland Esbjerg Denmark; ^6^ Department of Pediatrics Hospital of Southern Jutland Kolding Denmark; ^7^ Department of Clinical Genetics Odense University Hospital Odense Denmark; ^8^ Institute of Molecular Medicine University of Southern Denmark Odense Denmark

**Keywords:** adolescent, asthma, biomarker, child, surfactant protein D

## Abstract

**Background:**

Surfactant Protein D (SP‐D) is a pattern recognition molecule belonging to the family of collectins expressed in multiple human organ systems, including the lungs. Previous studies have shown that SP‐D levels in bronchoalveolar lavage samples decrease and serum levels increase in patients suffering from asthma, possibly due to a combination of induced SP‐D synthesis and decreased air–blood barrier integrity. The aims of this study were to investigate whether serum levels of SP‐D and common variants in the SP‐D gene were associated with asthma in adolescents and young adults.

**Methods:**

Prospective observational study including 449 adolescents and young adults (age 11–27 years) previously diagnosed with asthma during a 2‐year period from 2003 to 2005 (0–16 years). At follow‐up from 2016 to 2017, 314 healthy controls with no history of asthma were recruited. Serum SP‐D was analyzed on samples obtained at baseline as well as samples obtained at follow‐up. SP‐D genotyping was performed for rs721917, rs2243639, and rs3088308.

**Results:**

No differences were found in mean levels of sSP‐D and SFTPD genotype among subjects with *current asthma*, *no current asthma*, and *controls*. Serum SP‐D and SFTPD genotype were not associated with any clinical parameters of asthma. Furthermore, baseline sSP‐D was not associated with asthma at follow‐up.

**Conclusion:**

Serum surfactant protein D and common SP‐D gene variants were not associated with asthma in Danish adolescents and young adults with mild to moderate asthma. Serum surfactant protein D did not demonstrate any value as a clinical biomarker of asthma.

## Graphical abstract

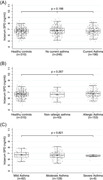

## 1 INTRODUCTION

Asthma is a common chronic airway disease defined by intermittent obstruction of the airways due to chronic inflammation.^1^ Clinical evaluation of patients suspected of asthma is based on airway symptoms, spirometry, and measures of airway hyperresponsiveness that are not always readily available in primary care nor feasible in preschool children. There is a pressing need for objective and readily available markers of airway inflammation in patients with asthma.

The etiology of asthma is complex and influenced by many factors, including genetic propensity, exposure to ubiquitous allergens, irritants, respiratory tract infections during early childhood, and altered airway microbiome.^2^, ^3^, ^4^, ^5^ Surfactant Protein D (SP‐D) is a pattern recognition molecule that belongs to the collectin family expressed in multiple human organ systems, including alveolar type II cells and Clara cells.^6^ SP‐D plays an important role in the innate immune system by binding bacteria, viruses, fungi, and parasites for clearance via opsonization in phagocytes, as well as aiding in the removal of allergens.^7^, ^8^, ^9^ Several experimental studies have indicated that SP‐D levels in broncho‐alveolar lavage fluid (BAL) decrease and serum levels increase in patients suffering from asthma possibly due to both induced SP‐D synthesis and decreased air–blood barrier integrity.^10^, ^11^, ^12^, ^13^ Recent clinical studies have revealed a correlation of serum SP‐D (sSP‐D) with asthma severity and a decreased BAL/serum SP‐D ratio supporting the hypothesis of leakage of degraded SP‐D to the circulation,^5^, ^14^, ^15^ whereas other studies have failed to demonstrate an association.^16^, ^17^

The surfactant protein D‐encoding gene (SFTPD) is located at the genomic position 10q22.2‐23.1.^18^ Three structural single nucleotide polymorphisms (SNP), rs721917, rs2243639, and rs3088308 have previously been linked with the circulating levels of SP‐D and pulmonary disease.^19^ In particular SNP in rs721917 has been shown to be associated with several chronic and acute respiratory diseases, although the data on the association to asthma have been conflicting.^20^, ^21^, ^22^

We intended to elucidate the clinical significance of SP‐D in childhood asthma in a real‐life setting. The aims of this study were to investigate if the serum level of SP‐D and SP‐D gene variants were associated with asthma in children, adolescents, and young adults, and whether serum SP‐D measured at the time of asthma diagnosis was associated with persistent asthma. We hypothesized that serum SP‐D was increased in subjects with persistent asthma and associated with asthma severity.

## 2 METHODS

### 2.1 Study design

Prospective follow‐up including 449 adolescents and young adults (11–27 years) from a closed cohort of children diagnosed with asthma during 2003–2005 (0–16 years) at all four pediatric outpatient clinics in the Region of Southern Denmark.^23^ At follow‐up during 2016–2017, an additional 314 healthy controls with no medical history of asthma or use of asthma medication, and within the same age range were recruited. Controls were recruited through a notice on the digital learning systems from the same schools as the origin of cases.

### 2.2 Baseline data collection at time of diagnosis

Baseline data were obtained from a structured database and biobank established prospectively during the baseline examination as previously described.^23^ The data were collected prospectively and consecutively by the treating staff and included a structured questionnaire‐based interview (regarding predisposition, medical history, medication use, and environmental exposures), clinical examination by a pediatrician, blood sampling, skin prick test, spirometry with bronchodilator test and exercise test where appropriate.

### 2.3 Follow‐up data collection

The follow‐up examination was performed by the research team which included medical doctors and nurses with experience within the field of pediatric asthma. For each participant, a structured questionnaire‐based interview based on the same items as the baseline questionnaire was completed. Subsequently, the subjects participated in a clinical examination, blood sampling, measurement of fractional exhaled nitric oxide (FeNO), spirometry with bronchodilator test, and mannitol provocation test. The subjects also completed questionnaires regarding asthma control (ACT, Asthma Control Test) and quality of life (Asthma Quality of Life Questionnaire).^24^, ^25^ Controls completed the same examination program as the cases except for mannitol provocation test and asthma questionnaires.

### 2.4 Serum surfactant protein D

Serum levels of SP‐D were measured by immunoassay as previously described.^26^ Serum SP‐D was analyzed on samples obtained at baseline as well as samples obtained at follow‐up.

### 2.5 SFTPD genotyping

DNA purification and genotyping were carried out by PentaBase Aps using Maxwell® 16 Blood DNA Purification Kit (Promega, AS1010) and nuclease resistant probes, EasyBeacons™, developed by PentaBase,^27^ in a 2‐step Real‐Time PCR, followed by melt analysis. SP‐D genotyping was performed for three single‐nucleotide variants conferring amino acid substitutions in the mature protein (rs721917, rs2243639, and rs3088308). Information on the SFTPD specific primers and probes (Table E1) and a detailed description of the method are provided in the online repository.

### 2.6 Definition of asthma

*Current asthma* at follow‐up was defined by recurrence or persistence of at least two of three symptoms: cough, wheeze, and shortness of breath (not triggered only by infection) within the last 12 months *and at least one of the following*: positive bronchodilator reversibility test (salbutamol/terbutaline) and/or positive mannitol test. Subjects were defined as having asthma if they had a history of asthma symptoms and daily use of inhaled corticosteroids (ICS), fixed combination of ICS, and long‐acting beta‐2‐adrenoreceptor agonists (LABA) or classical exercise‐induced asthma symptoms and clinical effect of inhaled beta‐2‐adrenoreceptor agonists. *Allergic asthma* was defined as having current asthma and concurrent allergic sensitization to ≥1 inhalant allergens. *Severe asthma* was defined as poor control despite maximal maintenance therapy (GINA step 4).^28^

### 2.7 Allergic sensitization

Specific immunoglobulin E (sIgE) at follow‐up was measured quantitatively using Single ImmunoCAP™ after an initial qualitative screening with ImmunoCap™ Phadiatop™ (Thermo Fisher Diagnostics Aps). The samples were tested for the presence of sIgE for 10 inhalant allergens (birch, timothy grass, mugwort, horse, dog, cat, *Dermatophagoides pteronyssinus*, *Dermatophagoides farinae*, *Alternaria*, and *Cladossporium*). Sensitization was defined as having sIgE ≥ 0.35 kU/l.

### 2.8 Clinical measures

*Lung function* was measured by spirometry (SpiroUSB, Carefusion Ltd), bronchodilator test was performed with salbutamol (Buventol Easyhaler™), and mannitol provocation testing was performed using the commercially available test kit (Osmohale™, Pharmaxis Ltd.). All measurements were performed according to generally accepted methods and criteria.^29^, ^30^, ^31^ Parameters obtained for statistical analysis were forced expiratory flow rate at one second (FEV1), forced vital capacity (FVC), FEV1/FVC, and forced expiratory flow 25%–75% (FEF25–75). We used the multi‐ethnic reference intervals for spirometry developed by the European Respiratory Society Task Force.^32^

Classification of the response to mannitol was performed in subjects with a positive test according to the cumulative dose of mannitol required to induce a 15% reduction in FEV1.^33^ Fractional exhaled nitric oxide (FeNO) was measured before spirometry using NIOX VERO™.

### 2.9 Statistical analysis

The distribution of data was evaluated visually using histograms and normal quantile‐quantile (QQ) plots. Because of a non‐normal distribution of observations of sSP‐D, approximation to normal distribution was achieved by logarithmic transformation (ln, the natural logarithm) before further statistical analysis. All reported values of sSP‐D and 95% confidence intervals were ln‐transformed.

The subjects were classified according to the clinical assessment at follow‐up: *current asthma*, *no current asthma*, and *controls* and subgroups of *allergic phenotype* and *response to mannitol*.

A regression‐based comparison using linear regression was used to test differences between ln sSP‐D as outcome and classification as exposure with an initial F‐test to test the overall difference between groups.

The relationships between ln(sSP‐D) and continuous clinical measures were assessed by estimating kernel‐weighted local polynomial regression. The association between sSP‐D as outcome and relevant exposure variables was estimated by linear regression models and presented by back‐transformed coefficients (e^ln(β)^) and confidence intervals (95%) with associated *p*‐values. The coefficients are interpreted as the fold increase per unit change in a continuous variable or the fold difference between levels of a binary explanatory variable.^34^ The longitudinal association of baseline sSP‐D with current asthma at follow‐up was estimated using logistic regression models.

All models were estimated using both a univariate model and a multivariate model to accommodate the uncertainty of potential confounders. In the multivariate models, we included age, sex, body mass index (BMI) (numeric), smoking status, and ethnicity, all factors previously reported to affect the constitutional levels of SP‐D.^35^, ^36^

The multivariate models assessing the association between sSP‐D and clinical measures of asthma were estimated on all study subjects and included asthma classification at follow‐up as an interaction term. Furthermore, all models were re‐estimated including smoking status, gender, and genotype in separate models as potential effect modifiers. Smoking status at follow‐up was defined as current active smoking whereas smoking status at baseline was defined as current parental smoking.

Analysis of genotype association was performed by initial testing of Hardy–Weinberg equilibrium of each SNP among the groups. Logistic regression models were applied to assess the association of asthma classification with genotypes.

To evaluate the importance of missing values on sSP‐D at follow‐up, subjects with a missing sSP‐D were assigned both the highest and the lowest measured value in the study population, and the main analysis was re‐estimated to assess whether conclusions changed.

All statistical analysis was performed using Stata 15 (StataCorp LCC) with the level of significance set to 0.05. Due to the risk of type one error caused by multiple testing findings should be interpreted as exploratory.

### 2.10 Ethics

The study was conducted in accordance with the Declaration of Helsinki approved by the Regional Scientific Ethical Committee of Southern Denmark (S‐20120093) and the Danish Data Protection Agency (95‐50819). Before enrollment, informed consent was obtained from each participant and from the parents of participants below 18 years of age.

## 3 RESULTS

### 3.1 Study population

The asthma diagnosis was confirmed in a total of 1014 children at baseline during 2003–2005 of whom 449/1014 subjects participated in the follow‐up examination during 2016–2017 (follow‐up rate, 0.443). 196 classified as having *current asthma* and 253 as having *no current asthma*. A comparison of the included subjects with the subjects lost to follow‐up is available in the online repository (Table E2). At follow‐up, 314 *healthy controls* were recruited. The basic and clinical characteristics of the study population stratified by classification at follow‐up are presented in Table 1. The groups had significant differences according to age, sex, BMI, ethnicity, and current smoking.

**Table 1:** Characteristics of the study population according to classification at follow‐up: *controls*, *no current asthma*, and *current asthma*

	Controls	No current asthma	Current asthma	
	(*n* = 314) %	(*n* = 253) %	(*n* = 196) %	
	(cases/total)	(cases/total)	(cases/total)	*p*‐value
Basic characteristics				
Gender ‐ female	61.8 (194/314)	39.9 (101/253)	46.4 (91/196)	<.001
Age ‐ mean (*SD*)	18.4 (4.5)	17.6 (4.4)	18.7 (4.1)	.030
BMI ‐ mean (*SD*)	22.2 (3.8)	22.9 (4.7)	23.2 (5.2)	.031
Ethnicity ‐ Caucasian	98.1 (308/314)	96.4 (244/253)	95.9 (188/196)	.312
Parental asthma^a^	10.5 (33/314)	29.6 (75/253)	33.2 (65/196)	<.001
Siblings ‐ median (IQR)	1 (1–2)	1 (1–2)	1 (1–2)	.480
Active smokers	5.4 (17/314)	8.7 (22/253)	10.7 (21/196)	.080
Symptom score (ACT)^b^				
Well‐controlled (score > 19)	–	94.0 (236/251)	77.4 (151/195)	
Poor controlled (score ≤ 19)	–	6.0 (15/251)	22.6 (44/195)	
Patient‐reported symptoms				
Hayfever	2.9 (9/314)	44.7 (113/253)	74.5 (146/196)	<.001
Eczema	2.2 (7/314)	11.1 (28/253)	27.7 (54/195)	<.001
Food allergy	1.0 (3/314)	5.1 (13/253)	10.2 (20/196)	<.001
Urticaria	0.6 (2/314)	4.3 (11/253)	13.8 (27/196)	<.001
Current medication^c^				
No treatment	–	87.0 (220/253)	11.7 (23/196)	
SABA only	–	13.0 (33/253)	19.9 (39/196)	
ICS low dose	–	–	29.6 (58/196)	
ICS moderate or ICS low dose + LABA	–	–	29.6 (58/196)	
ICS high or ICS moderate dose + LABA	–	–	9.2 (18/196)	
Add on (thiotropium/biologicals)	–	–	–	
Allergic sensitization				
Inhalant allergens	18.8 (58/309)	54.5 (134/246)	78.1 (153/196)	<.001
Food allergens	2.6 (8/309)	10.6 (26/246)	16.3 (32/196)	<.001

Abbreviations: ACT, Asthma Control Test; ICS, inhalant corticosteroids; LTRA, leukotriene receptor antagonists; *SD*, standard deviation; SABA, short‐acting beta2 agonists.

^a^ Medical history of asthma in ≥1 parent.

^b^ Not filled in by controls.

^c^ Self‐reported symptoms.

### 3.2 Association of sSP‐D with potential confounders

Significant relationships were found between sSP‐D and age (exp β, 0.97 [95% CI, 0.96–0.98]), female sex (exp β, 0.76 [95% CI, 0.70–0.82]), BMI (exp β, 0.97 [95% CI, 0.96–0.98]), and ethnicity (exp β, 1.58 [95% CI, 1.26–1.97]) but no significant association to current smoking (exp β, 0.95 [95% CI, 0.83–1.10]).

### 3.3 Association of sSP‐D with asthma at follow‐up

**Figure 1:**
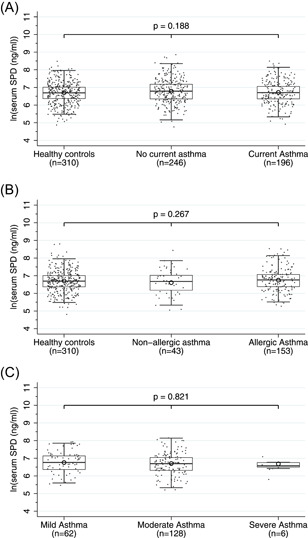
Boxplot presenting log‐transformed serum levels of surfactant protein D (SP‐D) (unadjusted) measured at follow‐up stratified by (A) classification at follow‐up, (B) allergic phenotype, and (C) asthma severity

All observations stratified by classification at follow‐up are presented in Figure 1A. No difference was found in mean levels of sSP‐D among subjects with *current asthma* 6.72 (95% CI, 6.64–6.80), *no current asthma* 6.79 (95% CI, 6.71–6.86), and *controls* 6.70 (95% CI, 6.65–6.76). Based on the findings regarding potential confounders, a multivariate model was estimated which did not change the conclusion.

When comparing mean levels of sSP‐D in subgroups, no differences were found according to allergic phenotype, *allergic asthma* 6.75 (95% CI, 6.66–6.84), *nonallergic asthma* 6.61 (95% CI, 6.42–6.80) (Figure 1B), and severity, *mild asthma* 6.76 (95% CI, 6.62–6.90), *moderate asthma* 6.70 (95% CI, 6.60–6.81) and *severe asthma* 6.68 (95% CI, 6.35–7.02) (Figure 1C).

**Figure 2:**
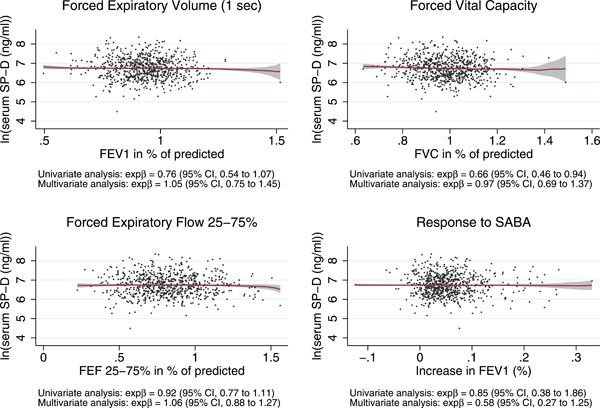
Association of serum SP‐D with parameters obtained from spirometry. Local kernel‐weighted mean of ln(SP‐D) on FEV1, FVC, FEF 25%–75% and response to SABA (% increase in FEV1). Shaded areas indicate 95% confidence bands. The linear slope coefficients are presented as back‐transformed coefficients (e^ln(β)^) with 95% confidence intervals from a univariate and multivariate model. The multivariate model includes adjustment for age, sex, BMI, active smoking, ethnicity, and asthma classification at follow‐up. FEF 25%–75%, forced expiratory flow 25%–75%; FEV1, forced expiratory flow rate at 1 s; FVC, forced vital capacity; SP‐D, surfactant protein D

**Figure 3:**
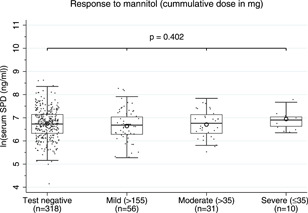
Boxplot presenting log‐transformed serum levels of surfactant protein D (SP‐D) (unadjusted) measured at follow‐up and stratified by response to mannitol. Mannitol test was only performed in subjects previously diagnosed with asthma: *current asthma* and *no current asthma*

No associations were found between sSP‐D and FEV1, FVC, FEF 25%–75% and response to SABA (Figure 2), nor to self‐reported atopic symptoms other than asthma, ACT‐score, FeNO, and allergic sensitization (data not shown). We found no association of sSP‐D with the outcome of mannitol test nor to the severity of the response to mannitol (Figure 3). There was a weak association of sSP‐D with FVC in the unadjusted model that diminished when age was included in the model.

No significant effect modification by gender, smoking status, genotype nor asthma classification at follow‐up was found in any model.

### 3.4 Association of baseline sSP‐D with current asthma at follow‐up

To investigate the longitudinal relationship of sSP‐D obtained at baseline with asthma status at follow‐up multivariate logistic regression models were estimated with asthma classification at follow‐up as response variable: *current asthma* vs *no current asthma*. The models included age, sex, BMI, ethnicity, and active parental smoking as covariates. No association of baseline sSP‐D was found with *current asthma* at follow‐up nor to FEV1, FVC, FEF 25%–75%, and response to mannitol.

### 3.5 Association of baseline sSP‐D with clinical measures obtained at baseline

The association of baseline sSP‐D with clinical measures obtained at baseline was evaluated and a weak association of baseline sSP‐D with FEV1 at baseline (age 5–16 years) was found in the adjusted model (exp β, 0.99 [95% CI, 0.99–1.00], *p* = .011). We found no association between baseline sSP‐D and FVC, FMEF, peak flow, atopic comorbidity, allergic sensitization, or blood eosinophils obtained at baseline.

### 3.6 Association STFPD gene variants with sSP‐D, asthma, and lung function

The frequencies of SNP's in the total number of genotyped subjects (*n* = 749) are presented in Table E3 in the supporting information. All SNP's were in Hardy–Weinberg equilibrium A univariate model and a multivariate model including all three SNP's were estimated to evaluate the association between genotypes and asthma. No association of STFPD variants, rs721917, rs3088308, and rs2243639 with current asthma (Table 2) nor to allergic asthma, FEV1, FVC, and FEF 25%–75% was found (data not shown). We found a strong association of rs721917 genotype with constitutional levels of sSP‐D both at baseline and follow‐up (Table 3) and a high degree of tracking comparing the baseline levels of sSP‐D with sSP‐D at follow‐up (Figure E4).

**Table 2:** Association of SFTPD polymorphisms and asthma at follow‐up (2016–2017)

		“Current asthma” vs. “Controls”		“Current asthma” vs. “No current asthma”	
		(*n* = 502)		(*n* = 442)	
SNP	Genotype	OR (95% CI)	*p*‐value	OR (95% CI)	*p*‐value
rs2243639	CC	Ref.	–	Ref.	–
	CT	1.06 (0.71–1.57)	.787	0.90 (0.60–1.37)	.633
	TT	0.77 (0.45–1.33)	.343	0.83 (0.46–1.49)	.537
rs3088308	AA	Ref.	–	Ref.	–
	AT	0.83 (0.48–1.45)	.518	0.93 (0.52–1.67)	.801
	TT	6.21 (0.69–56.05)	.104	5.06 (0.56–45.68)	.149
rs721917	AA	Ref.	–	Ref.	–
	AG	1.33 (0.89–2.00)	.164	1.11 (0.73–1.69)	.627
	GG	1.11 (0.66–1.87)	.687	1.23 (0.71–2.15)	.459

Note: Unadjusted logistic regression models are presented with odds ratios (ORs), 95% confidence intervals (CIs), and related *p*‐values.

**Table 3:** Associations of sSPD with SFTPD variants *rs2243639*, *rs3088308*, and *rs721917*

			Univariate model		Multivariate model	
	Variant	Genotype	expβ (95% CI)	*p*‐value	expβ (95% CI)	*p*‐value
Follow‐up						
	rs2243639	CC	Ref.	–	Ref.	–
		CT	1.04 (0.95–1.13)	.404	0.86 (0.77–0.95)	.003
		TT	1.13 (1.01–1.28)	.035	0.76 (0.65–0.88)	<.001
	rs3088308	AA	Ref.	–	Ref.	–
		AT	0.75 (0.66–0.84)	<.001	0.83 (0.74–0.94)	.003
		TT	0.65 (0.42–1.00)	.048	0.77 (0.50–1.18)	.222
	rs721917	AA	Ref.	–	Ref.	–
		AG	0.79 (0.73–0.86)	<.001	0.74 (0.67–0.82)	<.001
		GG	0.68 (0.61–0.76)	<.001	0.60 (0.52–0.70)	<.001
Baseline						
	rs2243639	CC	Ref.	–	Ref.	–
		CT	1.01 (0.90–1.13)	.848	0.89 (0.78–1.02)	.091
		TT	1.08 (0.93–1.27)	.309	0.83 (0.68–1.00)	.056
	rs3088308	AA	Ref.	–	Ref.	–
		AT	0.75 (0.64–0.88)	<.001	0.79 (0.67–0.94)	.007
		TT	0.63 (0.40–1.00)	.050	0.69 (0.43–1.09)	.113
	rs721917	AA	Ref.	–	Ref.	–
		AG	0.85 (0.76–0.95)	.004	0.82 (0.72–0.94)	.005
		GG	0.76 (0.66–0.89)	<.001	0.74 (0.60–0.90)	.003

*Note*: The models are estimated for sSPD both measured at follow‐up and baseline. The linear slope coefficients are presented as back‐transformed coefficients (e^ln(β)^) with 95% confidence intervals (CIs). The multivariate models include all three SFTPD variants,

### 3.7 Missing values

To investigate the importance of missing values of sSP‐D at follow‐up (missings, *n* = 11) on the association with asthma at follow‐up, subjects with a missing sSP‐D were assigned both the highest and the lowest measured value in the study population, and the main analysis was subsequently re‐estimated with no change of the conclusion at follow‐up. When the subjects were assigned the lowest measured value of sSP‐D, the mean levels of sSP‐D in subjects with current asthma were 6.72 (95% CI, 6.64–6.80), no current asthma 6.72 (95% CI, 6.64–6.81), and controls 6.68 (95% CI, 6.62–6.74). When the subjects were assigned the highest measured value of sSP‐D, the mean levels of sSP‐D in subjects with current asthma were 6.72 (95% CI, 6.64–6.80), no current asthma 6.83 (95% CI, 6.75–6.91), and controls 6.72 (95% CI, 6.67–6.78).

## 4 DISCUSSION

### 4.1 Main findings

In the present study, we found no association of serum SP‐D with asthma in adolescents and young adults with predominantly mild to moderate asthma. We found no association of sSP‐D with lung function, FeNO, allergic sensitization, response to SABA, and mannitol. Baseline sSP‐D at the time of asthma diagnosis during childhood was not associated with persistent asthma into adolescence and young adulthood. Furthermore, we evaluated three single nucleotide polymorphisms, rs721917, rs3088308, and rs2243639, in the SP‐D gene and found no association with asthma. To our knowledge, no previous data on the association of sSP‐D with asthma in children, adolescents, and young adults have been published.

### 4.2 Interpretation of the findings

Our results are in agreement with previous clinical studies reporting data on sSP‐D in patients with mild to moderate asthma.^16^ In contrast, recent studies have found that sSP‐D is associated with asthma severity and the degree of small airway dysfunction^14^, ^15^ and that SP‐D concentrations in BAL samples were reduced and serum concentrations elevated in patients with severe treatment‐resistant asthma.^5^ A reduction in BAL SP‐D and concomitant increase in sSP‐D has also been observed in patients with COPD and in subjects exposed to tobacco smoke^37^, ^38^ and has been proposed to reflect nonspecific inflammation within the distal airways and alveoli, suggesting an impaired endothelial barrier and altered permeability which allows leakage of both full‐size and degraded SP‐D into serum.^5^ This hypothesis is supported by a study by Sin et al that demonstrates an increased level of airway SP‐D messenger RNA, indicating that the reduced level of BAL SP‐D is not caused by diminished synthesis but rather leakage into serum.^37^ On the contrary, a study recently reported a decreased level of sSP‐D in subjects with aspirin‐exacerbated respiratory disease.^39^

In the present study, we found no indications of leakage of SP‐D into serum in a population of adolescents and young adults who were diagnosed with asthma at four pediatric outpatient clinics in the Region of Southern Denmark during 2003‐2005. The majority of the population was receiving medication according to GINA Steps 1–3 with varying symptom control assessed by Asthma Control Test, whereas only six patients with *current asthma* had severe disease defined as poor asthma control despite maximal maintenance therapy (GINA Step 4) and acceptable adherence to treatment. It may be speculated that the differences in the local environment in the airways of our cases compared with controls are minimal and without any influence on the endothelial barrier of the airways. It is well‐known that severe asthma represents a distinct phenotype characterized by an altered airway microbiome associated with neutrophilic airway inflammation, a more pronounced expression of Th2 signature molecules, and reduced lung function.^40^, ^41^, ^42^, ^43^

Experimental studies have demonstrated that asthma is associated with increased expression of SP‐D in the airways, and that SP‐D seems to exert negative feedback on interleukin (IL)‐13, thus promoting key features in asthma such as eosinophil infiltration, production of IgE, and airway hyperresponsiveness.^44^ SP‐D gene deficiency induces hyper‐eosinophilia, increased levels of IL‐5 and IL‐13, and a lowered IFN‐gamma to IL‐4 ratio, an immune response that is reversible by treatment with SP‐D.^45^, ^46^ The design of this study does not allow definite conclusions on the underlying molecular mechanisms in the airway tissue. It is noted, however, that we did not observe any indirect evidence of these immune pathways on the clinical parameters in our study.

Previous studies investigating the heritability of sSP‐D found that the genetic variant, rs721917, accounts for 39% of the variation in sSP‐D,^36^ strongly affecting the constitutional levels of sSP‐D and the degree of protein multimerization.^22^, ^47^ Furthermore, is has been shown that the rs721917 genotype varies significantly according to ethnicity.^35^ Several previous studies on SP‐D in relation to pulmonary disease do not include SFTPD variations in the analysis. This gives rise to some uncertainty whether associations previously reported are explained by a causal relationship or a result of differences in genotypes among the groups. This underlines the importance of evaluating this genotype in studies on sSP‐D.

### 4.3 Strength and limitations

The major strengths of this study are the large sample size including comprehensive characterized subjects in a real‐life clinical setting and the ability to include relevant confounders in the analysis. An important drawback of the study is the lack of airway samples, such as bronchoalveolar lavage samples and bronchial biopsies, which limits our ability to investigate the molecular mechanisms in the target tissue related to changes in the airway microbiome and immunological responses. Furthermore, this study did not address previous findings of impaired structural integrity of SP‐D in patients with severe asthma.^5^

This study included only six subjects with *current asthma* and severe disease defined as poor asthma control despite maximal maintenance therapy (GINA Step 4) and acceptable adherence to treatment. It is therefore only possible to conclude that we did not find any association of sSP‐D with increased asthma severity but not to draw any conclusion regarding patients with severe asthma.

## 5 CONCLUSION

Serum SP‐D and three common single nucleotide polymorphisms in the Surfactant Protein D gene, rs721917, rs3088308, and rs2243639, were not associated with asthma in Danish adolescents and young adults with mild to moderate asthma. Serum SP‐D did not demonstrate any value as a clinical biomarker of asthma in adolescents and young adults with mild to moderate asthma.

## AUTHOR CONTRIBUTIONS

Susanne Halken and Arne Høst were responsible for the baseline study as a whole from design to conduction of the study and data collection. Lone Agertoft, Benjamin Hoffmann‐Petersen, Susanne Halken, and Arne Høst designed and initiated the follow‐up study. The clinical examinations were performed by Benjamin Hoffmann‐Petersen, Raymond Suffolk, Jens Jakob Herrche Petersen, and Thomas Houmann Petersen. Benjamin Hoffmann‐Petersen was responsible for uniformity and consistency of the study across the hospitals and supervised all examinations. Grith Lykke Sorensen was responsible for laboratory analysis of SP‐D. Charlotte Brasch‐Andersen facilitated the genetic analysis and interpretation. Benjamin Hoffmann‐Petersen was responsible for data collection, data analysis, and writing the manuscript. All co‐authors have contributed substantially to the interpretation of the data, provided crucial intellectual input, and approval of the final draft of the manuscript.

## Supporting information

Supporting information.

## Data Availability

The standard terms for research projects and the Danish Act on Processing of Personal Data define the rules of data sharing and will be followed. Data used for the manuscript may be obtained in anonymous form after application for permission to the regional Danish Data Protection agency (https://www.datatilsynet.dk/english/).
