# Supplementary material for: Association of serum surfactant protein D and SFTPD gene variants with asthma in Danish children, adolescents, and young adults
Source: Immun Inflamm Dis. 2021 Nov 15;10(2):189–200. doi: 10.1002/iid3.560 (PMC8767520; doi:10.1002/iid3.560)
Supplement: Supplementary file 1 — Supporting information. [file IID3-10-189-s001.pdf]

Supporting information

Methods

DNA purification and SFTPD genotyping

DNA purification and genotyping were carried out by PentaBase Aps, Odense, Denmark. Genomic DNA was purified from EDTA whole blood de-frozen at room temperature (RT) and 3 ml was transferred to bluecap tubes. In two independent cycles an equal volume (3 ml) buffer A (1.6 M Sucrose, 5 mM $MgCl_2$, 1 % Triton X-100, and 1 mM TRIS-HCl pH 7.5) was added and subjected to vortex and centrifugation (10 minutes, 3000 rpm, RT). Supernatant was discharged by decantation. Subsequent pellets were resuspended in 200 µl H_2O.

Purification was performed with Maxwell® 16 Blood DNA Purification Kit (Promega, AS1010) on a Maxwell® 16 instrument (Promega) and eluted in 350 µl elution buffer. SP-D genotyping was performed for 3 single-nucleotide variations conferring amino acid substitutions in the mature protein (rs721917, rs2243639 and rs3088308). Genotyping was performed with nuclease resistant probes, EasyBeacons™, developed by PentaBase (1), in a 2-step Real-Time PCR, followed by melt analysis. Five µl eluated DNA was analyzed in rs721917 simplex assay (single determination, validity of assay determined by independent duplicate analysis of > 20 samples) and 5 µl eluated DNA analyzed in rs2243639 + rs3088308 multiplex assay (single determination, validity of assay determined by independent duplicate analysis of > 30 samples). Information on the SFTPD specific primers and probes are provided in table E1.

Thermocycling and melt analysis was performed on a MyGo Pro qPCR instrument (IT-IS Life Science) applying the following conditions: 2 min of initial activation of the hotstart taq-polymerase at 95°C, followed by 60 cycles of a 2-step PCR with a 15 sec denaturation step at 94°C and 30 sec extension step at 60°C and ending with a pre-melt hold (95°C for 10 sec) and melting from 37-97°C with 0.1°C/s.

Table E1. Primer sequences.

	Forward primer:	Reverse primer:	Probe:
**rs721917**	GCTACACATGACCAGGGTGC (1000 nM)	CTGTTCACCTCTCCAGGC (100 nM)	PentaOrange CCCACAGAACAACGCCC OrangeQuencher (1000 nM)
**rs2243639**	GCTCTTTCCACTGCTCAC (80 nM)	AGGGAGAGCGAGGTGTC (800 nM)	PentaGreen CTGCGTTTCCAGGGACTC GreenQuencher (600 nM)
**rs3088308**	ATGCTCAGGAAAGCAGC (120 nM)	CCATTTACGGAGGCACAGC (1200 nM)	PentaYellow AGCGTGGAGAGGCCAAC YellowQuencher (600 nM)

Results

Table E2. Characteristics of subjects included in the follow-up examination compared to subjects lost to follow-up.

	**Included at follow-up** (n=449) % (cases/total)	**Lost to follow-up** (n=565) % (cases/total)	chi2/t-test p-value
Sex - female	42.8 (192/449)	32.7 (185/565)	0.001
Age - Mean (sd)	6.2 (4.1)	6.5 (4.1)	0.242
Hospital †			
Esbjerg	17.1 (77/449)	15.9 (90/565)	0.681
Kolding	17.4 (78/449)	20.2 (114/565)	
Odense	40.3 (181/449)	38.4 (217/565)	
Sonderborg	25.2 (113/449)	25.5 (144/565)	
Current atopic symptoms			
Hayfever	27.6 (124/449)	28.8 (163/565)	0.665
Eczema	23.8 (107/449)	26.9 (152/565)	0.265
Food Allergy	3.8 (17/449)	5.8 (33/565)	0.133
Urticaria	3.3 (15/449)	6.0 (34/565)	0.048
Current medication			
SABA	86.0 (386/449)	82.8 (468/565)	0.173
ICS	70.4 (316/449)	65.8 (372/565)	0.124
LTRA	1.6 (7/449)	1.8 (10/565)	0.795
Allergic sensitization			
Inhalant allergens	52.9 (220/416)	53.7 (277/516)	0.808
Food allergens	34.8 (145/417)	33.7 (175/520)	0.720

Notes: †, Hospital on which the baseline examination was performed
Abbreviations: sd, standard deviation; SABA, shortacting beta2 agonists; ICS, inhalant corticosteroids; LTRA, leukotrienreceptorantagonists

Table E3. Frequencies of SFTPD single-nucleotide polymorphisms.

SNP Location	**Alleles**	Observed frequency N	Observed frequency %	**Genotypes**	Observed frequency N	Observed frequency %	Expected frequency (a) N	Expected frequency (a) %	HW Equilibrium Test p-value
rs2243639	C	882	0.5888	CC	256	0.3418	259.68	0.3467	
(10:79941966)	T	616	0.4112	CT	370	0.4940	362.67	0.4842	
missense variant				TT	123	0.1642	126.66	0.1691	
	Total	1498	1.0		749	1.0	749.01	1.0	0.5811
rs3088308	A	1393	0.9299	AA	650	0.8678	647.66	0.8647	
(10:79938112)	T	105	0.0701	AT	93	0.1242	97.67	0.1304	
missense variant				TT	6	0.0080	3.67	0.0049	
	Total	1498	1.0		749	1.0	749	1.0	0.2271
rs721917	A	873	0.5828	AA	260	0.3471	254.44	0.3397	
(10:79946568)	G	625	0.4172	AG	353	0.4713	364.24	0.4863	
missense variant	Total			GG	136	0.1816	130.40	0.1741	
		1498			749	1.0	749.08	1	0.3988

Abbreviations: N, numbers; HW, Hardy-Weinberg; A, adenine; C, cytosine; T, thymine; G, guanine.

Figure E4.

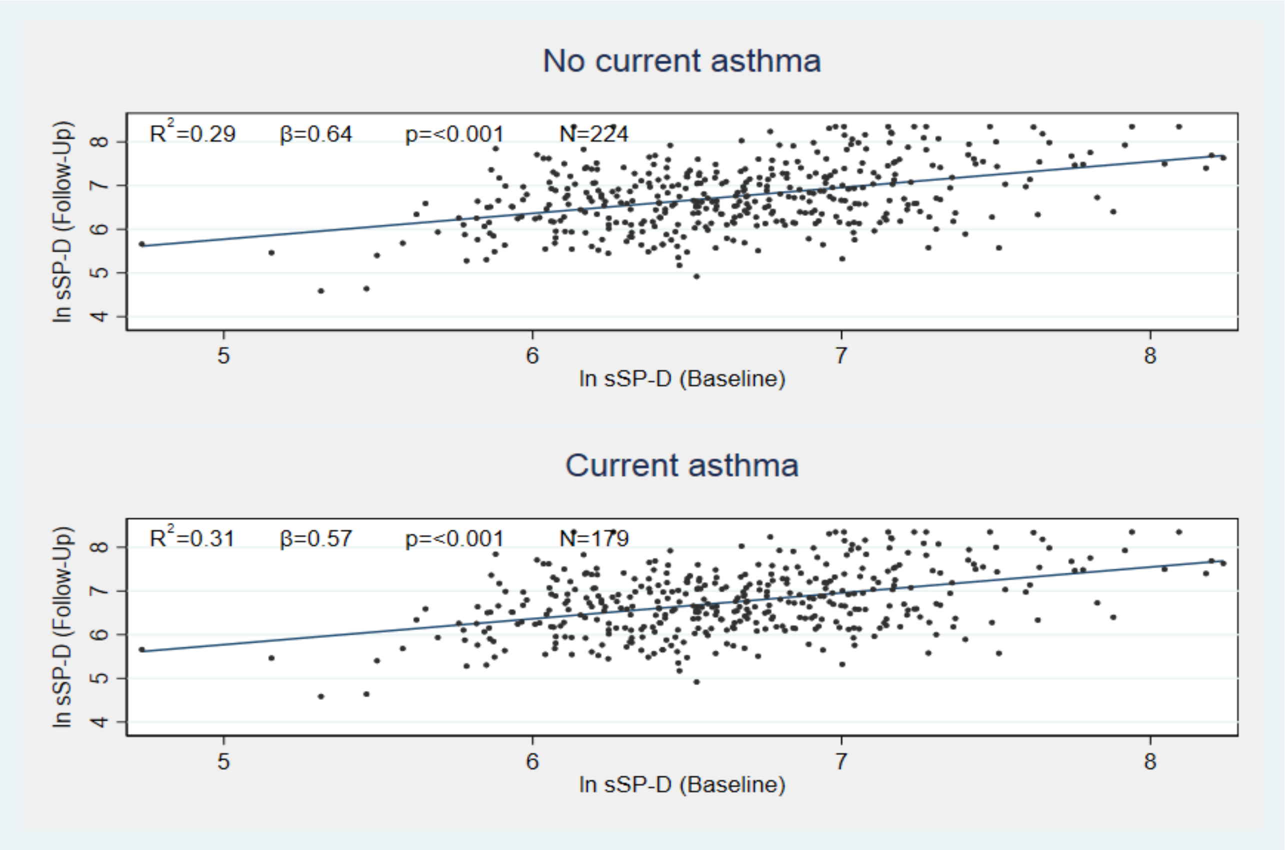

References

1. Christensen UB. EasyBeacons[TM] for the Detection of Methylation Status of Single CpG Duplets. In: Marx A, Seitz O, editors. *Molecular Beacons: Signalling Nucleic Acid Probes, Methods, and Protocols*. Totowa, NJ: Humana Press 2008: 137–160.
